# Vitamin D and the Liver—Correlation or Cause?

**DOI:** 10.3390/nu10040496

**Published:** 2018-04-16

**Authors:** Jeremy T. Keane, Harendran Elangovan, Rebecca A. Stokes, Jenny E. Gunton

**Affiliations:** 1 Centre for Diabetes, Obesity & Endocrinology, The Westmead Institute for Medical Research (WIMR), Westmead, Sydney, NSW 2145, Australia; jeremytkeane@gmail.com (J.T.K.); rebecca.stokes@sydney.edu.au (R.A.S.); 2 The Garvan Institute of Medical Research, The University of New South Wales (UNSW), Darlinghurst, Sydney, NSW 2010, Australia; hari338@hotmail.com; 3 Faculty of Medicine and Health, The University of Sydney, Westmead Hospital, Westmead, Sydney, NSW 2145, Australia

**Keywords:** vitamin D, VDR, liver fibrosis, NAFLD

## Abstract

Vitamin D is becoming increasingly accepted as an important physiological regulator outside of its classical role in skeletal homeostasis. A growing body of evidence connects vitamin D with hepatic disease. This review summarises the role of vitamin D in liver homeostasis and disease and discusses the therapeutic potential of vitamin D-based treatments to protect against hepatic disease progression and to improve response to treatment. While pre-clinical experimental data is promising, clinical trials around liver diseases have mostly been under-powered, and further studies will be required to clarify whether vitamin D or vitamin D analogues have beneficial effects on liver disease.

## 1. Introduction

In addition to its classical role in bone and calcium homeostasis, there is an expanding volume of data regarding associations between vitamin D deficiency and several medical pathologies, including multiple sclerosis, Alzheimer’s disease, myopathy and cancer. Liver disease has also been strongly linked to vitamin D deficiency, and the development of the vitamin D receptor (VDR) knockout (VDRKO) murine model has greatly increased our understanding of vitamin D’s role in the liver. 

Vitamin D deficiency is common in chronic liver diseases [1]. However, despite several impressive in vitro and in vivo studies using human liver cell lines and animals and the ease of access to vitamin D supplements and analogues, patient clinical data thus far remains inconclusive. Studies involving vitamin D interventions in liver disease, like other diseases, have tended to be small and have not yielded definitive results. This review aims to update the reader on the functions of vitamin D in the liver and analyse any future roles for vitamin D treatment. 

## 2. Vitamin D and the Vitamin D Receptor

### 2.1. Vitamin D, Its Active Metabolites, and Causes of Vitamin D Deficiency

Vitamin D is a fat-soluble hormone which is obtained from exposure to sunlight, diet, and from some health supplements. Dietary sources include oily fish, shitake mushrooms and fortified foods which can include milk and cereals, and supplements [2]. Sunlight may be responsible for up to 90% of the requirement of vitamin D in most people [3]. In the skin, 7-dehydrocholesterol changes upon sunlight exposure (ultraviolet B irradiance) to form pre-vitamin D3, which is then converted to vitamin D3 [4]. 

Vitamin D deficiency is typically defined as levels below which the risk of osteomalacia and rickets increases [5]. However, the expanding body of evidence linking low vitamin D levels with non-skeletal pathologies raises important questions about optimal levels necessary for general health and well-being. 

Systemic vitamin D status is assessed by measuring the level of 25-hydroxyvitamin D (25D). Population studies focusing on latitude, sunlight exposure, and vitamin D fortification are important, and have increased our understanding of vitamin D deficiency. However, fluctuations in sunlight exposure and the content of dietary vitamin D provide challenges in assessing population vitamin D consumption [6]. Moreover, the emerging data highlighting the role of VDR polymorphisms in vitamin D responsiveness further confounds population-based recommendations pertaining to vitamin D intake.

### 2.2. Vitamin D Uptake, Storage, and Metabolism

**Figure 1:**
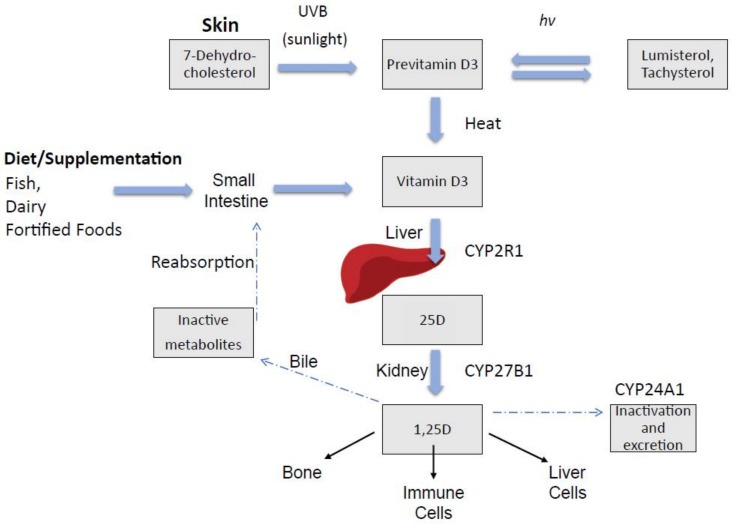
Vitamin D endogenous synthesis and metabolism. Endogenous vitamin D synthesis occurs primarily through sunlight exposure which produces pre-vitamin D3. It is hydroxylated in the liver and then in the kidney, producing 1,25D (1,25 dihyroxyvitamin D), the physiologically active form of vitamin D which acts in target sites in bone and immune cells, as well as liver cells. Abbreviations: CYP (cytochrome P450), UVB (ultraviolet B), hν (denotes photochemical reaction).

Vitamin D in circulation is bound to the vitamin D-binding protein (DBP) [7,8], with approximately 88% of 25D being bound to DBP [9]. DBP may regulate the eventual delivery of 25D and the active 1α,25(OH)2D3 (1,25D) ligand to tissues, making it available to activate the transcription factor, VDR, and influence downstream gene transcription [10], as shown in Figure 1.

### 2.3. Vitamin D Receptor

First identified in 1974 [11], the vitamin D receptor (VDR) is the receptor to which the biologically active form of vitamin D (1,25D) binds to exert its effects. The VDR is a nuclear hormone receptor with three distinct regions: a DNA-binding N-terminal dual zinc finger domain, a C-terminal domain that binds to 1,25D, and a large, non-binding region that links the two functional domains. The binding of 1,25D to VDR causes conformational changes within the receptor that allow it to interact with a dimeric partner, usually the retinoid X receptor (RXR). The liganded heterodimer then binds to vitamin D response elements (VDREs) in the DNA [12], facilitating gene transcription. VDR homodimers may also bind DNA to regulate gene expression in the absence of RXR. Unusually, VDR may also affect gene transcription in its unliganded state [13]. In addition to mediating the actions of 1,25D, synthetic VDR analogues, as well as the bile acid, lithocholic acid (LCA), may function as secondary ligands of the receptor [14]. VDR is expressed in virtually every tissue of the body, though its expression levels vary significantly between different cells [15]. 

### 2.4. VDR in the Liver 

Hepatocytes isolated from humans, rats, and mice express little or no VDR mRNA and protein [16,17]. On the other hand, non-parenchymal cells, including hepatic stellate cells (HSCs), sinusoidal endothelial cells, and Kupffer cells (KCs), strongly express VDR [18], and these probably mediate vitamin D effects in the non-diseased state. However, levels of VDR in hepatocytes increase with inflammation, giving a broader site for potential targets. Levels also increase in HSCs and KCs with inflammation, and several immune functions of VDR described in other tissues may also be involved in liver disease (Table 1).

## 3. Viral Hepatitis C and Vitamin D

### Hepatitis C, Vitamin D and VDR

Despite a decline in the incidence of hepatitis C virus (HCV) and the recent improvement in antiviral treatments, HCV remains a significant disease, with 160 million people worldwide chronically infected [25]. HCV-related complications account for over 700,000 deaths each year [26]. Vitamin D deficiency is common in HCV patients [27,28].

Research indicates a potentially beneficial role for vitamin D signalling in the clinical response to HCV therapy, with a possible role in virus persistence and clearance. Studies examining 25D levels in chronic HCV patients have reported an association between low 25D levels and reduced rates of sustained virological response (SVR) [29,30]. Cell culture experiments have indicated that vitamin D may reduce HCV viral load. Yano et al. studied the effects of vitamin D on HCV RNA replication in cell culture, finding that vitamin D2 inhibited HCV RNA replication at concentrations greater than those found in the circulation [28]. It also synergises with interferon (IFN) in cell culture to inhibit HCV RNA replication [31], which may be explained by an IFN-sparing effect [32] and increased autophagic genes, such as G-protein coupled receptor 37 (GPR37) [31]. 

A few studies have investigated the relationship between the vitamin D status of patients with chronic hepatitis C and disease outcomes. A Sicilian study of one hundred and ninety-seven patients with genotype 1 (G1) and 49 healthy subjects found that low vitamin D is associated with severe fibrosis and low SVR in patients on IFN and ribavirin therapy [30].

Studies have examined vitamin D related gene polymorphisms and their potential roles in impacting treatment outcomes**.** The VDR bAt haplotype (haplotype of BsmI, ApaI, and TaqI alleles) is associated with failure to respond to Peg-IFN/ribavirin therapy [33,34]. Furthermore, polymorphisms in the VDR gene are also associated with treatment failure and poor sustained virologic response (SVR) [35]. 

Whether vitamin D supplementation impacts the outcome of HCV treatment remains an area of contention. A retrospective review of Italian patients who were treated with IFN and ribavirin for recurrent HCV post-transplantation found that patients who had concomitant treatment with vitamin D for bone disease had significantly higher SVR rates [36]. Two small, randomised studies investigating the effect of 2000 IU/day vitamin D3 supplementation in patients on an IFN and ribavirin regimen showed vitamin D supplementation increases the rate of SVR in patients infected with HCV genotypes 1–3 [37,38]. In the first study, Abu-Mouch and colleagues studied seventy-two patients with chronic HCV genotype 1, with the treatment group receiving Peg-α-2b IFN (1.5 μg/kg per week) plus ribavirin (1000–1200 mg/day) together with a vitamin D dose of 2000 IU per day, while the control group received the same antiviral treatment without vitamin D supplementation. They found that the vitamin D-treated group had an increased SVR rate [37]. Nimer and colleagues showed similar results [38]. However, the patient sample of 50 was too small to draw definite conclusions. In contrast, a study by Ladero and colleagues involving 41 chronic HCV patients who were diagnosed with vitamin D deficiency did not find any significant effect of vitamin D treatment on the viral load of HCV or on biochemical markers of necroinflammation [39]. Another study also found no improvement in patient outcomes when vitamin D levels were corrected before initiating Peg-IFN/ribavirin therapy in previous non-responders with HCV genotype 1 or 4 [40].

Although in vitro data shows that vitamin D decreases viral replication, further clinical studies are needed to determine whether HCV patients can benefit from treatment with vitamin D or its analogues. It is also important to note that HCV may reduce 25D levels by directly affecting vitamin D production, which can complicate studies focusing upon 25D and HCV correlations. Clark and colleagues found that HCV reduces the production of the vitamin D precursor, 7-dehydrocholesterol [41]. The relationship between low 25D levels and reduced SVR could also be explained by the correlation between low SVR and fibrosis severity. The reduced expression of liver enzymes involved in the hydroxylation of vitamin D also results from reduced liver function in disease, which may at least explain the reduced 25D levels observed in chronic hepatic disease. However, as reported by Petta and colleagues, patients with mild fibrosis also have reduced 25D levels, meaning that reduced liver function alone is unlikely to fully explain lower 25D levels [30] Vitamin D-binding protein is also reduced in chronic liver disease [42], reducing the distribution of vitamin D to tissues, as well as further complicating patient studies. Therefore, further work is required to determine a role for vitamin D or vitamin D analogue treatment in HCV patients. 

We note that, as yet, there have been no trials examining the effect of vitamin D with the new highly-active HCV therapies. With their very high levels of SVR, these studies are perhaps unlikely to be conducted given the large numbers needed to achieve adequate statistical power. It seems reasonable, given lack of harm and possible benefit, to recommend replacing vitamin D in HCV patients who are vitamin D deficient, preferably before, or at the commencement of, anti-viral therapy. 

## 4. Vitamin D and Hepatitis B 

### Vitamin D, VDR Polymorphisms and Hepatitis

Like HCV, vitamin D deficiency is common in people with HBV infection, and 25D levels are associated with higher viral replication rates in patients with chronic HBV infection [43,44]. However, not all studies have found this—Zhao and colleagues did not find a relationship between vitamin D and HBV DNA levels [45], and Chan et al. reported that the association was not statistically significant [46]. More studies examining the course of 25D levels over the natural progression of chronic HBV infection may provide further information. 

Boglione and colleagues found the TT genotype (homogenous for T allele) of the CYP27B1+2838 single nucleotide polymorphism (SNP) was associated with positive treatment outcomes in patients receiving Peg-IFN treatment [47]. Associations were also reported between clinical phenotypes and VDR polymorphisms in hepatitis B virus (HBV) carriers [48], as well as between the Taq1 and Fok1 polymorphisms, and virologic outcomes in a Chinese study. A meta-analysis of 15 published studies, including 4218 cases and 2298 controls, reported the FokI polymorphism, specifically the genotypes, FF and Ff, and the allele, F, as a potential risk factor for HBV infection [49]. In this analysis, the authors found no association between VDR TaqI, ApaI and BsmI polymorphisms and HBV infection risk [49]. However, the effect of VDR gene polymorphisms upon the course of HBV infection may also differ between different ethnicities and warrants further investigation. The use of VDR pharmacogenomics to predict treatment outcomes or provide personalised therapy remains a promising concept.

Vitamin D deficiency has been associated with a poor response to active hepatitis B immunisation in patients with chronic kidney disease [50]. Also, Mahamid and colleagues reported an association between normal vitamin D levels and spontaneous hepatitis B surface antigen sero-clearance [51]. This small retrospective study of 53 patients with chronic, inactive hepatitis B and spontaneous hepatitis B surface antigen sero-clearance found that among the 53 patients who exhibited long-term hepatitis B antigen sero-clearance, 44 patients had normal levels of 25D, while nine patients had below normal levels (<30 ng/mL) [51]. On the other hand, Jhorawat and colleagues reported no statistically significant differences in vitamin D levels in dialysis patients between responders and non-responders to the HBV vaccination [52]. Further studies, particularly in immunocompetent patients, will be required to determine the role of vitamin D in HBV vaccination.

## 5. Vitamin D and Non-Alcoholic Fatty Liver Disease (NAFLD)

NAFLD is a spectrum of diseases, progressing from simple steatosis, through to non-alcoholic steatohepatitis (NASH), fibrosis, and cirrhosis [53,54]. Obesity is a significant risk factor, and the prevalence of the disease globally has increased rapidly. NAFLD is strongly associated with type 2 diabetes (T2DM) and increased cardiovascular risk. Indeed, NAFLD is believed to represent the hepatic manifestation of the metabolic syndrome, with insulin resistance highlighted as a key molecular signature in its pathogenesis. Hypocaloric diets and exercise both reduce steatosis, which highlights the importance of lifestyle factors in both the development and treatment of the disease.

There are currently no drug treatments for NAFLD licensed by the Food and Drug Administration. Lifestyle interventions such as weight loss and moderate exercise improve markers of apoptosis and insulin sensitivity as well as liver fat content. Among the most promising treatment strategies to date are those relating to the treatment of T2DM, which may have an indirect effect on NAFLD through improvements in insulin resistance and glycaemic control. 

### 5.1. NAFLD Pathogenesis

NAFLD pathogenesis involves an increase in hepatic lipids resulting from increased synthesis and influx of fatty acids to the liver, leading to hepatic steatosis. Adipocyte infiltration to the liver causes increased inflammatory signalling and hepatocyte lipotoxicity [55]. Increased oxidative stress, resulting from free radicals generated during beta-oxidation of fatty acids, the release of inflammatory cytokines by hepatic stellate and Kupffer cells (KCs) and increased ER stress also contributes to disease progression [55]. Both apoptosis and necrosis pathways may become activated, leading to hepatic stellate cell (HSC) activation, collagen deposition and hepatic fibrosis.

VDR and 25D have been proposed as regulators in adipose tissue and may, therefore, be involved in NAFLD pathogenesis. Indeed, vitamin D deficiency is more common in people with NAFLD compared to healthy individuals [56]. Both VDR and 25-hydroxyvitamin D 1α-hydroxylase (CYP27B1) mRNA are expressed in mouse and human adipocytes [57,58], as are several other vitamin D-related genes [59]. Vitamin D influences adipokine production and the inflammatory response in adipocytes [60]. In a large study of two patient cohorts, serum 25D levels correlated with the adiponectin (an adipocyte with potent anti-inflammatory properties) concentration, independent of patient BMI (body mass index) [61]. As adipocyte dysregulation is a feature of NAFLD, and fat cells are vitamin D responsive, the possibility of vitamin D being involved in NAFLD seems mechanistically plausible. 

### 5.2. Vitamin D and NAFLD Progression

Roth and colleagues, using a rat model of NAFLD, found that vitamin D deficiency resulted in increased hepatic inflammation. The authors found that a diet deficient in vitamin D increased toll-like receptor (TLR) activity as well as insulin resistance [20]. Interestingly, macrophage VDR deletion increased insulin resistance, indicating that different VDR-related immune functions may overlap with other metabolic roles [62]*.* In an apparent contradiction to other studies, Bozic and colleagues reported that hepatocyte VDR is induced early in NAFLD and may be a detrimental factor in disease progression [63]. The authors postulated that VDR regulates hepatic lipid metabolism genes and may instead promote liver steatosis [63].

Human studies have suggested a role for vitamin D in the development of NAFLD. Like other liver diseases, clinical studies have tended to report an inverse association between 25D and the histologic severity of NAFLD. Liangpunsakul and Chalasani reviewed over 6800 patients and found that patients with elevated alanine transaminase (ALT) had lower vitamin D levels than those with less elevated ALT, even when adjusting for other factors [64]. Also, Targher and colleagues, in a small study of 60 patients with NAFLD, found that patients with NAFLD had a marked decrease in the concentration of serum 25D, and that decreased serum 25D levels were correlated with the histological severity of NAFLD including hepatic steatosis and fibrosis [65], which is corroborated by other studies [56,66]. The findings of these studies were brought into context by a study by Lee and colleagues, who studied 82 patients with NAFLD over two months, providing nutritional advice to assist weight loss [67]. The authors found that weight loss increased serum vitamin D levels and improved the metabolic parameters in NAFLD [67]. Whether the repletion of vitamin D levels had any mechanistic role in the improvements in the NAFLD phenotype remains to be elucidated. Interestingly the study also found that weight loss was more effective than vitamin D supplementation in increasing serum vitamin D levels in NAFLD patients. In contrast, Bril and colleagues studied 239 patients, dividing them into three groups according to plasma 25D levels (normal >30 ng/mL; insufficiency 20–30 ng/mL; deficiency <20 ng/mL) [68]. After matching for clinical parameters, no significant differences were found among the groups in terms of insulin sensitivity, the amount of liver fat, or the severity of liver inflammation or fibrosis [68]. 

Consistent with the inconclusive retrospective data, conflicting reports have been published examining whether vitamin D supplementation has any effect on the progression and severity of NAFLD. Barchetta et al. [69], in a study of 65 patients with type 2 diabetes and NAFLD, found that oral vitamin D supplementation of 2000 IU per day over 24 weeks did not improve hepatic steatosis as detected by the hepatic fat fraction and fatty liver index.

Sharifi and colleagues, in a small study with 27 patients on high dose supplementation, and 26 people on placebo, found that treatment of 50,000 IU every two weeks for four months decreased inflammatory markers, including serum C reactive protein, in NAFLD patients compared to those in the placebo group [70]. However, ultrasound investigation did not reveal any differences in hepatic injury [71]. In another study, Amiri et al. studied 120 patients for 12 weeks and found no differences by liver ultrasound between patients taking calcitriol compared to a placebo [71]. The short concomitant weight loss program followed by all patients tended to mask any potential effects. 

In addition to its potential effects on immune and inflammation processes, studies are required to investigate whether vitamin D reduces oxidative stress in NAFLD. To date, a few animal studies have indicated that vitamin D may have some benefit, such as one study which found that vitamin D reduced TNF-α and oxidative stress markers in the adipose tissue of rats on a high fat diet [21]. Human studies are again lacking in this area and have been largely inconclusive. At the present time, there is no convincing data to support the use of vitamin D to improve outcomes in NAFLD. Nevertheless, people with a deficiency should be treated, given the well-established musculoskeletal benefits.

## 6. Fibrosis of the Liver and Vitamin D

Liver fibrosis is a process characterised by the accumulation of extracellular matrix, as a consequence of chronic liver injury or disease, including viral infection, alcoholic liver disease, and NAFLD, which may progress to cirrhosis, liver failure, and hepatocellular carcinoma [72]. The activation of hepatic stellate cells (HSCs), a process which follows liver injury, is a crucial driver of fibrosis, whereby quiescent, vitamin-A-storing cells transdifferentiate into proliferative, fibrogenic myofibroblasts in an impaired wound response. Extracellular signals, including those from the extracellular matrix and inflammatory cells, promote HSC activation from various mechanisms. Pro-fibrogenic cytokines, including transforming growth factor-β (TGF-β), platelet-derived growth factor (PDGF) and vascular endothelial growth factor (VEGF), among others, are involved in the liver’s inappropriate wound repair response which results in the inappropriate production of collagen and tissue scarring [72,73,74]. TGF-β, for example, is produced following liver injury and mediates wound repair through the apoptosis of damaged cells and tissue regeneration [75]. However, impaired wound repair occurs when this process is dysregulated, leading to inappropriate TGF-β-driven cell damage and apoptosis [75].

### VDR in Liver Fibrosis

The cell-specific expression pattern of VDR suggests that the liver could be responsive to vitamin D during liver fibrosis through its non-parenchymal cells, in particular, HSCs. Abramovitch and colleagues showed, in 2011, that ligation of VDR in HSCs inhibited their proliferation, and activation and reduced thioacetamide (TAA)-induced liver fibrosis in rats [76]. Further, Ding and colleagues revealed that VDR ligation in activated hepatic stellate cells has anti-fibrotic effects, which are mediated through a VDR/SMAD3/TGF-β signalling loop [77]. The authors also carried out genetic studies in mice which resulted in spontaneous liver fibrosis when one or both *Vdr* alleles were knocked out, with more severe fibrosis occurred in *Vdr^−/−^* animals [77]. 

The VDR-SMAD-HSC signalling loop initially proposed by Ding and colleagues has since been verified through various studies, including a recent report that VDR ligation suppresses HSC activation in cultured human HSCs, thereby inhibiting the development of fibrosis and also hepatocellular carcinoma (HCC) [78]. These findings, taken together with the correlation between low vitamin D serum levels and the severity of liver fibrosis [30], suggest that not only may vitamin D deficiency contribute to liver fibrosis, but also that vitamin D supplementation may manifest anti-fibrotic effects in liver disease.

Recently, Wahsh and colleagues, using a TAA animal model of fibrosis, found that the VDR agonist, calcipotriol, modulates fibrogenic pathways and mitigates liver fibrosis in-vivo when injected via the intraperitoneal route at a dose of 80 ug/kg [79]. The authors found that calcipotriol improved liver function and reduced liver inflammation, necrosis and fibrosis percentage, as detected by histological analyses. ELISA techniques also demonstrated that calcipotriol reduces hepatic collagen-1-alpha-1 (Col1a1), Tissue Inhibitor of Metalloproteinase (TIMP), and TGF-β1 protein as well as the activity of the TGF-β-SMAD pathway, the most potent fibrogenic mechanism in the liver [79]. Belifuss et al. found that vitamin D represses fibrogenic TGF-β signalling in human hepatic stellate cells, both receptor-dependently and independently [80], which suggests that the genomic effects of VDR outlined by Ding and colleagues are not the only anti-fibrotic mechanisms through which vitamin D may act. For the reasons outlined above, 25D plasma levels and distribution may be altered in chronic liver fibrosis, and thus, any human studies must be carefully designed to reflect this. Therefore, further studies are required to elucidate the pathways involved and whether these may be appropriately translated into clinical application. 

## 7. Vitamin D Signalling in Hepatocellular Carcinoma

### 7.1. Hepatocellular Carcinoma and Vitamin D

Hepatocellular carcinoma (HCC) is a primary cancer of the liver and is one of the leading causes of cancer-related death worldwide [81]. The role of VDR in HCC has received considerable attention for several reasons. Firstly, polymorphisms of VDR have been associated with HCC in cirrhosis patients [82]. Secondly, serum vitamin D is a purported prognostic parameter in HCC, with reduced survival rates in patients with low serum 25D. Vitamin D treatment has been associated with anti-proliferative activity in several HCC cell lines [78,83]. Consistent with these observations, vitamin D analogues, including EB1089, CB 1093 and MART-10, have shown promise as potential therapeutic agents for HCC [84]. Indeed, EB1089, a synthetic VDR ligand diminished the development of HCC in C3H/Sy mice, a strain predisposed to spontaneous liver carcinogenesis [85].

### 7.2. HCC and Vitamin D Signalling

Hepatic stellate cells are known to be a major factor in fibrogenesis and are also suspected to be involved in HCC pathogenesis through the same mechanisms. VDR, which is significantly enhanced in activated stellate cells, may be important in HCC development through stellate cell activation. Recently, Duran and colleagues found that the vitamin D receptor inhibits liver cancer as well as fibrosis through a process involving p62/SQSTM1 ligation of the VDR [78]. Through isolation of cultured HSCs in HCC patients, the authors found that p62 loss in the stroma is a common feature of HCC. They also used HSC-specific p62-deficient mice to identify p62 as an HSC repressor and a non-cell autonomous tumour suppressor, which functions through VDR/RXR [78]. Interestingly the authors also showed that p62 is overexpressed in HCC cells. Whether this is a tumour promoting effect or compensatory response to facilitate VDR anti-proliferative activity is currently unknown.

### 7.3. Patient Data

A phase II clinical trial of the vitamin D analogue EB1089 (seocalcitol) found that of 33 patients with inoperable HCC, two patients showed a complete response and 12 showed disease stabilisation, while 19 patients did not respond [86]. Unfortunately, the authors did not assess the patients for vitamin D deficiency prior to the study, and the small sample size and lack of control group make it difficult to draw conclusions. Further human studies that are adequately-powered and include a control group are needed.

The utility of vitamin D as an adjunct to existing chemotherapeutic or surgical models requires further investigation. Given the role of the fibrogenic phenotype as a tumorigenic niche which dampens the fibrotic response, in particular by modulating HSC activation [78], or modulating cancer immunology [87] through the use of VDR ligands, these models are potential future therapeutic strategies for HCC. 

## 8. Vitamin D and Autoimmune Liver Disease

The role of vitamin D in autoimmune disorders such multiple sclerosis has been well-established. Also, there has been an increased incidence in several autoimmune diseases in more Northern climates, which is believed to be associated with vitamin D levels. Lower levels of vitamin D have been reported in several autoimmune diseases, such as multiple sclerosis [88], rheumatoid arthritis (RA), and diabetes mellitus and autoimmune thyroid disease [3,88,89]. However, the functions of vitamin D in autoimmune liver diseases, such as primary biliary cholangitis (PBC), autoimmune hepatitis, and primary sclerosing cholangitis (PSC), have yet to be defined. Here, we examine the role of vitamin D as an immunomodulator and its potential role in autoimmune liver diseases.

Vitamin D and VDR function in many components of the immune system, including monocytes, macrophages, natural killer (NK) cells, T lymphocytes, B lymphocytes, and dendritic cells (DC). Mahon and colleagues identified over 100 genes that are the targets of 1,25-dihydroxyvitamin D (1,25D) in mature T helper (Th) cells, with 57 genes being downregulated and 45 genes being upregulated by 1,25D treatment [90]. In addition, vitamin D represses Th1 and Th17 responses and increases the production of T-regulatory cells, interleukin-10 (IL-10) and NK cells, all processes that are likely involved in the pathogenesis of autoimmunity [91,92,93]. Moreover, Boglione and colleagues [48] highlighted its potential role in regulating B cell proliferation, differentiation and antibody production. As well as being expressed in T and B lymphocytes, VDR is present in other important cells involved in innate immunity, including neutrophils, macrophages and dendritic cells. Adorini et al. showed that 1,25D has a dampening effect on the adaptive immune response through the inhibition of DC cell differentiation, maturation and activation, promoting the development of a tolerogenic phenotype [94]. 

### 8.1. Primary Biliary Cholangitis

Primary biliary cholangitis (PBC), also known as primary biliary cirrhosis, is an autoimmune liver disease characterised by the destruction of intrahepatic bile ducts. Female patients make up approximately 90% of patients [95]. As vitamin D is a known modulator of immune function and osteoporosis is common in PBC [96], several studies have been published investigating its role in PBC [97,98].

Genome-wide association studies (often termed GWAS) in PBC patients have revealed that VDR Bsm1 and Taq1 polymorphisms are associated with susceptibility to advanced fibrosis or cirrhosis [99]. Also, recent studies have established that low vitamin D levels are correlated with increased markers of hepatic dysfunction in patients with PBC [100,101]. 

The mechanisms through which vitamin D may function in PBC have been examined. Firrincieli and colleagues found that deficiency in VDR promotes cholestatic liver injury in mice by increasing the disruption of biliary epithelial cell junctions [102]. A study by Kempinska-Podhorodecka and colleagues also suggested that VDR signalling could be involved in the pathogenesis of PBC through reduced SOCS1 expression, leading to increased cytokine levels [103]. These findings are consistent with emerging data that suggests that cholangiocytes manifest immune regulatory properties that promote the development of a tolerogenic hepatic phenotype. As these cells are bathed in a reservoir of VDR ligands, such as LCA, further work to elucidate the roles of the VDR axis in cholangiocyte physiology is warranted.

Ursodeoxycholic acid (UDCA), which is the mainstay of treatment for PBC, may work through the VDR. D’Albert et al. investigated the regulation of cathelicidin, a peptide with antimicrobial actions [19], in the biliary epithelium [104]. They found that in cultured biliary epithelial cells (BECs), cathelicidin expression is induced by UDCA via the VDR and suggested that combining UDCA with vitamin D may increase its therapeutic efficacy in inflammatory biliary disease [104]. This mechanism may explain findings from the study by Guo et al. which determined that baseline vitamin D levels predict the response to UDCA therapy [100]. 

Currently, more research is needed to ascertain whether low levels of vitamin D or VDR polymorphisms affect the development, progression or severity of PBC. As noted earlier, low levels of vitamin D are a known consequence of liver disease. In addition, reduced vitamin D absorption occurs in intestinal malabsorption conditions, including cholestasis [105,106,107]. Therefore, further studies are required to fully explain the cause–effect relationship between vitamin D status and PBC and determine whether improving vitamin D status can result in better disease outcomes in these patients.

### 8.2. Autoimmune Hepatitis

Autoimmune hepatitis (AIH) is characterised by histological chronic hepatitis, elevated serum transaminase levels and usually, a good response to immunosuppressants [108]. Similar to PBC, it mostly affects women (approximately 75%) [109], however, unlike the PBC phenotype, patients with AIH usually respond quickly to corticosteroid treatment, highlighting a potential variance in immune and inflammatory mechanics [110]. Studies focusing on the role of vitamin D in AIH are lacking. However, associations between the Fok polymorphism of VDR and AIH have been described [97,110].

### 8.3. Primary Biliary Sclerosis 

Data investigating Primary Biliary Sclerosis (PBS) and vitamin D are also lacking. However, given the modulatory effects of vitamin D upon both the immune and inflammation axes, possible roles for vitamin D and vitamin D analogues may exist. Vitamin D may affect the inflammatory phenotype involved in PSC by affecting TNFα-mediated downregulation of CD28 in liver-infiltrating T cells [111].

## 9. Vitamin D and Hepatic Transplantation

Vitamin D deficiency is highly prevalent at the time of hepatic transplantation [112]. Redaelli and colleagues found, in a rodent model of liver transplantation, that the combination of 1,25 D and cyclosporine reduced the severity of acute cellular rejection and extended the survival of liver allografts when compared with treatment of cyclosporine alone [113]. Moreover, Gregori and colleagues used a mouse islet allograft model to demonstrate that the use of VDR ligands in combination with mycophenolate mofetil increases transplantation tolerance in association with increased CD4+CD25+ regulatory cells [22]. Similarly, Xing and colleagues [114], who reported that calcitriol reduces the incidence of acute cellular rejection in liver transplants, speculated that T-regulatory cells are involved. A more recent study, which used human Jurkat clone E6-1T cells, found that 1,25(OH)2D3 controls the differentiation of Treg/Th17 cells by acting on the VDR/phospholipase C/TGF-β1 pathway [23]. On the other hand, Zhang and colleagues found that vitamin D may reduce hepatocyte allograft rejection by reducing hepatocyte apoptosis [115]. More research is required to assess a role for vitamin treatment in liver transplant patients (Table 2).

## 10. Conclusions

**Figure 2:**
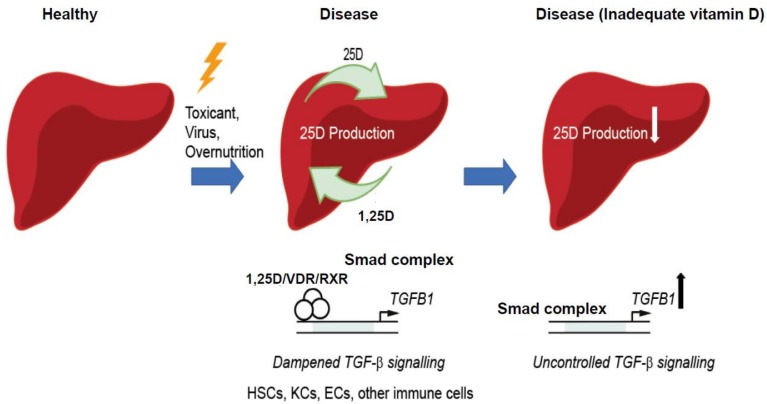
Proposed mechanisms of vitamin D signalling in chronic liver disease. Adequate vitamin D production in disease may reduce fibrosis development through effects of VDR in HSCs or other cells. Abbreviations: HSCs (hepatic stellate cells), KC (Kupffer cells), ECs (endothelial cells), RXR (retinoid X receptor), TGF-β (transforming growth factor-β), *TGFB1* (gene encoding TGF-β1), Smad (mothers against decapentaplegic homologue (Drosophila).

Animal studies, in particular knockout studies, have helped to greatly increase our understanding of the mechanisms through which vitamin D functions in liver disease. However, despite the promising in vitro and in vivo data produced over the past two decades, further refinement in cell-specific knockout models and pathway analyses, permitting the study of VDR in HSCs, KCs, endothelial cells, as well as infiltrating immune cells, will hopefully shed additional light on the molecular topology of VDR signalling in the spectrum of liver diseases (Figure 2).

The following questions remain in relation to the use of vitamin D in patients with liver disease. Is vitamin D deficiency merely a consequence of liver disease, or does deficiency cause or worsen it? Also, what are the long-term effects of vitamin D supplementation in liver disease? We also need to further elucidate the off-target effects of taking vitamin D at supraphysiological levels for disease treatment, or the use of potent VDR analogues, particularly because VDR is expressed throughout the body. The relative importance of VDR polymorphisms and biomarker expression in predicting responses to VDR-based strategies is also important. Despite the current uncertainties, there is undoubtedly still therapeutic potential for vitamin D or its analogues in liver disease. Larger randomised controlled trials are required, as well as further studies into the use of vitamin D as an adjunct to existing therapies, such as in HCC and viral hepatitis.

Definitive clinical data for liver fibrosis patients with vitamin D or VDR agonist treatment are lacking. We are not aware of any long-term randomised controlled trials in humans. Pharmacological parameters, including the optimal dosing route/delivery vectors and bioavailability considerations given the histologic, and functional distortions of the cirrhotic microenvironment would be key considerations and limitations of potential trial design.

More research is required to assess a role for vitamin D treatment in liver transplant patients. However, as it is established that patients requiring liver transplantation have an increased risk of fracture [117], and transplant recipients on immunosuppressive or corticosteroid therapy have an even further increased risk of osteoporosis, we recommend treating any recipients who have low vitamin D levels to normal levels. Lowered risk of rejection may be a benefit of that treatment. Until we have more reliable long-term data regarding vitamin D and vitamin D analogues in liver disease, it would be premature to recommend their use for the sake of liver health alone. For now, it is prudent to adhere to the Endocrine Society’s guidelines for those with liver failure or malabsorption and screen for vitamin D deficiency in patients with chronic or severe liver disease and correct vitamin D levels to normal for bone health [116]. 

## Figures and Tables

**Table 1:** Potential direct and indirect mechanisms of effect of vitamin D in liver diseases.

Cell	Process	Potential Roles in Liver Disease
Hepatocyte	Antiviral effect through ↓ HCV replication (mechanisms unclear)	Improved SVR in HCV
Hepatic Stellate Cells	↓ fibrotic markers through ↓ SMAD3 binding to promoter sites, with associated ↓ proliferation and ↓ HSC activation	↓ fibrosis in viral hepatitis or fibrosis patients ↓ HCC proliferation
Macrophage	↑ Cathelicidin through activation of TLR and increased VDR expression [19], ↓ TNF-α, IL-16	HCV and HBV response and clearance
Cytokines	↓ TNFα, IL-4, IL-6 andTLR repressed [20]	↓ liver inflammation NAFLD
Adipocytes	↓ oxidative stress; ↓ TNFα and inflammatory markers in adipose tissue [21]	↓ inflammation in NAFLD
T cells	Increases Treg cells or Treg differentiation [22,23] Activates naïve T cells [24]	*↓* acute cellular rejection liver transplantation Anti-proliferative effect in HCC
Cholangiocytes	Protection of integrity of biliary epithelial cell junctions; ↑ Cathelicidin (in primary cultured cells)	Improvements in primary biliary cholangitis autoimmune hepatitis and primary sclerosing cholangitis; enhanced effect of ursodeoxycholic acid (UDCA) treatment

↓ = decreased, ↑ = increased. Abbreviations: HCC (hepatocellular carcinoma), HCV (hepatitis C virus), HSC (hepatic stellate cell), IL (interleukin), NAFLD (non-alcoholic fatty liver disease), SMAD (mothers against decapentaplegic homologue), SVR (sustained virological response), TGF (transforming growth factor), TLR (toll like receptor), TNF (tumor necrosis factor), Treg (regulatory T cells), VDR (vitamin D receptor).

**Table 2:** Selected intervention studies in liver disease and study parameters.

Study (Ref.)	Disease	Basal 25D	Study Deficiency Definitions	Treatment, Duration, Number of Patients	Key Outcomes
Abu-mouch et al. [37]	HCV (Hepatitis C virus)	Measured, 21% were severely 25D deficient, 59% of patients were 25D insufficient and 20% had sufficient levels	Not as per Endocrine Society definitions * Severe deficiency set as <12 ng/mL	Vitamin D3 (2000 IU/day + anti-viral treatment (*n* = 36) versus anti-viral treatment alone (*n* = 36), 4 weeks, total of 72 patients	Improved sustained virological response (SVR) in chronic hepatitis C,21% of the patients had severe deficiency, 59% had an insufficiency, and 20% had sufficient vitamin D levels
Bitetto et al. [36]	HCV	Measured, 71% patients were deficient at baseline	As per Endocrine Society	Anti-viral treatment + vitamin D3 800 IU/day (*n* =15), anti-viral treatment alone (*n* = 27), 48 weeks	Vitamin D supplementation improved the probability of SVR
Amiri et al. [71]	NAFLD (non-alcoholic fatty liver disease)	Measured, baseline 25D levels were equal among treatment groups before study (10 ± 0.63, 9.9 ± 0.64, 9.9 ± 0.64)	Not defined	25 mg calcitriol (*n* = 37), 25 mg calcitriol + 500 mg calcium carbonate (*n* = 37), placebo (*n* = 36) daily, 12 weeks	Improved fasting blood glucose; additional calcium provided better outcomes
Barchetta et al. [69]	NAFLD	Measured, at baseline, 67% had deficiency	Not as per Endocrine Society; deficiency set at <50 nmol/L	Vitamin D 2000 IU daily; 24 weeks; treatment (*n* = 29) placebo (*n* = 36)	Vitamin D did not improve hepatic steatosis
Dalhoff et al. [86]	hepatocellular carcinoma (HCC)	No details, dose adjusted according to calcium levels	Not defined	EB1089 (seocalcitol), 33 patients treated; 2 years or until death	Possible tumour stabilisation in inoperable HCC

* Vitamin D deficiency is defined by the Endocrine Society as a 25D level below 20 ng/mL, and vitamin D insufficiency as a 25D level of 21–29 ng/mL [116].
